# Pseudomyxoma peritonei induced by low-grade appendiceal mucinous neoplasm accompanied by rectal cancer: a case report and literature review

**DOI:** 10.1186/s12893-019-0508-6

**Published:** 2019-04-25

**Authors:** Shili Ning, Yanliang Yang, Chen Wang, Fuwen Luo

**Affiliations:** Department of General Surgery, The Second Hospital of Dalian Medical University, Zhongshan Road, Shahekou District, Dalian City, Liaoning Province People’s Republic of China 116023

**Keywords:** Pseudomyxoma peritonei, Appendiceal mucinous neoplasm, Rectal cancer, Diagnosis, Treatment

## Abstract

**Background:**

Pseudomyxoma peritonei (PMP) is a disease involving the peritoneum characterized by the production of large quantities of mucinous ascites. PMP has a low incidence, is difficult to diagnose, and has a guarded prognosis. PMP induced by low-grade appendiceal mucinous neoplasm is extremely rare, and PMP accompanied by rectal cancer is even rarer.

**Case presentation:**

We present a unique case of a 70-year-old male with PMP induced by low-grade appendiceal mucinous neoplasm accompanied by rectal cancer. The patient’s clinical, surgical, and histologic data were reviewed. The patient had persistent distended abdominal pain without radiating lower back pain, abdominal distension for 1 month, and no exhaustion or defecation for 4 days. A transabdominal ultrasound-guided biopsy was performed on the first day. The patient received an emergency exploratory laparotomy because of increased abdominal pressure. We performed cytoreductive surgery, enterolysis, intestinal decompression, special tumour treatment and radical resection of rectal carcinoma. The postoperative course was uneventful. The postoperative histological diagnoses were PMP, low-grade appendiceal mucinous neoplasm and rectal medium differentiated adenocarcinoma. At the 1-year follow-up visit, no tumour recurrence was observed by computed tomography (CT). We also performed a literature review.

**Conclusions:**

We should be aware that PMP can rarely be accompanied by rectal cancer, which represents an easily missed diagnosis and increases the difficulty of diagnosis and treatment. Additionally, there are some typical characteristics of PMP with respect to diagnosis and treatment.

## Background

Pseudomyxoma peritonei (PMP) is a disease involving the peritoneum characterized by the production of large quantities of mucinous ascites, which progressively fill the peritoneal cavity [1]. It has been 176 years since Carl Rokitansky first described an appendiceal mucocele in 1842. However, the origin, pathology, treatment, prognosis, and even the very definition remain controversial [2, 3]. The primary lesion typically originates from adenoma, mucinous appendicular adenocarcinoma or ovarian tumours. Dissemination occurs by the rupture of the lesion, which releases tumour cells into the abdominal cavity. The progressive accumulation of mucinous ascites occasionally produces partial or complete obstructive symptoms [1, 2]. Because PMP lacks specific clinical manifestations, it is difficult to diagnose before surgery [4].

PMP induced by low-grade appendiceal mucinous neoplasm (LAMN) accompanied by rectal cancer is extremely rare. There are a few reports of PMP accompanied by rectal cancer [5–8]. To the best of our knowledge, no cases of PMP induced by low-grade appendiceal mucinous neoplasm (LAMN) accompanied by rectal cancer have been reported. In this paper, we share our experience with this rare presentation.

## Case presentation

A 70-year-old male was admitted to our hospital for “abdominal pain, abdominal distension for 1 month, and no exhaustion or defecation for 4 days” as the chief complaint on April 10, 2017. He had no fever, nausea or vomiting.

**Fig. 1:**
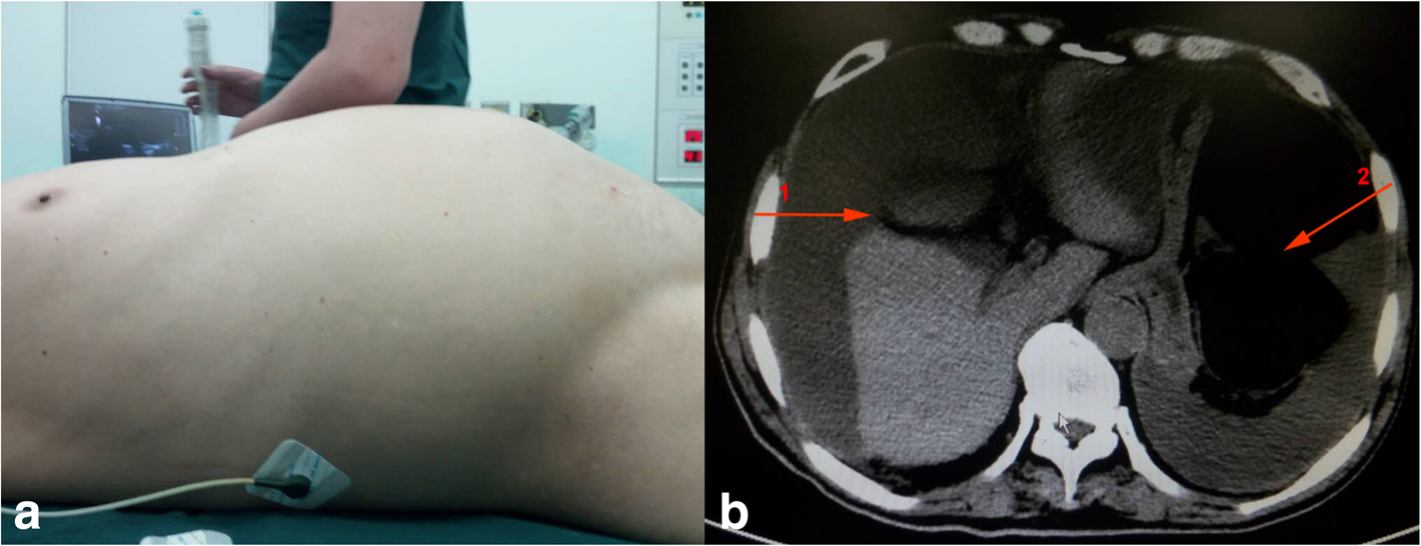
Physical examination and abdominal computed tomography scans. Physical examination revealed abdominal distension (**a**). Abdominal computed tomography images of the patient revealed peritoneal effusion (arrow 1) and bowel dilatation (arrow 2) (**b**)

The physical examination revealed abdominal distension (Fig. 1a), full abdominal tenderness and weak bowel sounds (1 beat/min). The following laboratory data were observed: WBC: 9.02 × 10^9^/L, NET%: 78.90%, and CEA: > 60.00 μg/L. No obvious electrolyte, coagulation or liver biochemistry abnormalities were noted.

**Fig. 2:**
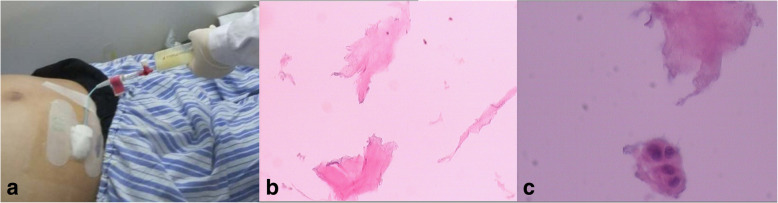
Transabdominal ultrasound-guided biopsy of the patient and microscopic appearance of the mucus. Yellow, jelly-like mucus was extracted via transabdominal ultrasound-guided biopsy (**a**). Mesothelial cells, fibrous tissue and lymphocytes with mild atypia were noted (**b**, **c**)

A CT scan of the abdomen revealed peritoneal effusion and bowel dilatation (Fig. 1b). The admitting diagnoses that were investigated were acute intestinal obstruction and abdominal effusion. On the first day, a transabdominal ultrasound-guided biopsy was performed, and a characteristic yellow jelly-like mucus containing microscopic mesothelial cells, fibrous tissue and lymphocytes with mild atypia was extracted (Fig. 2a-c). Therefore, PMP was suspected.

**Fig. 3:**
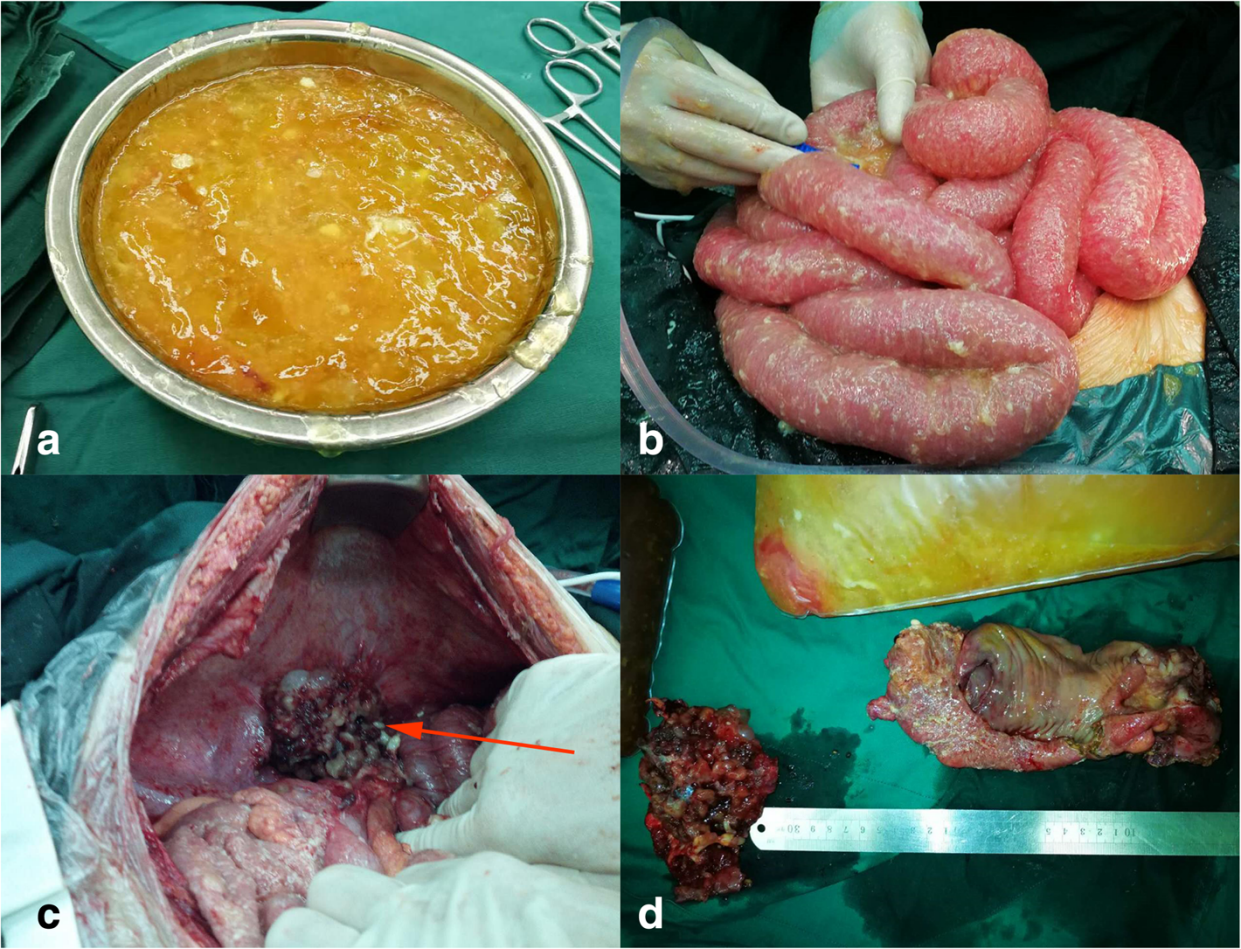
Findings of laparotomy. Large quantities of yellow, jelly-like mucus (**a**). The patient’s small intestine and colon were expanded (**b**). A hard mass (arrow) was identified on the ileocecal junction (**c**). Appendiceal tumour with gangrenous rupture and rectal tumour (**d**)

Operation: Because the patient complained of increasing abdominal distension and his abdominal pressure reached 35 mmHg, he underwent an emergency exploratory laparotomy. A significant amount of yellow, jelly-like mucus (approximately 5000 mL) was found during the operation (Fig. 3a). Numerous metastases were noted on the omentum and mesenteric root. After removing the mucus, we identified a hard mass measuring 10 cm × 15 cm with an unclear boundary and an abundant blood supply on the ileocecal junction (Fig. 3c). After carefully separating the appendix, the gangrenous rupture of the ileocecal tumour was observed, and the appendiceal lumen was interlinked with the abdomen. The patient’s small intestine and colon were expanded, but the colon’s expansion was more obvious, corresponding to low intestinal obstruction (Fig. 3b). Considering that explanations other than paralytic intestinal obstruction caused by the significant accumulation of intraperitoneal mucus might be plausible, we further explored the pelvic cavity. A hard mass measuring 4 × 5 cm with an unclear boundary infiltrating the rectal muscle layer was identified in the upper rectum (Fig. 3d). The peritoneal cancer index (PCI) was estimated intraoperatively, and the aggregative score of 13 abdominopelvic regions reached 20. We performed cytoreductive surgery (CRS), enterolysis, intestinal decompression and special tumour treatment to remove the lesions and relieve the obstruction as much as possible. Although some residual cancer remained, there was no nodule larger than 2.5 mm in diameter. Thus, we performed CC1 cytoreduction on the patient. Radical resection of the rectal carcinoma was also performed because the patient had PMP accompanied by rectal cancer.

The postoperative course was uneventful. The patient was discharged on postoperative day 15.

**Fig. 4:**
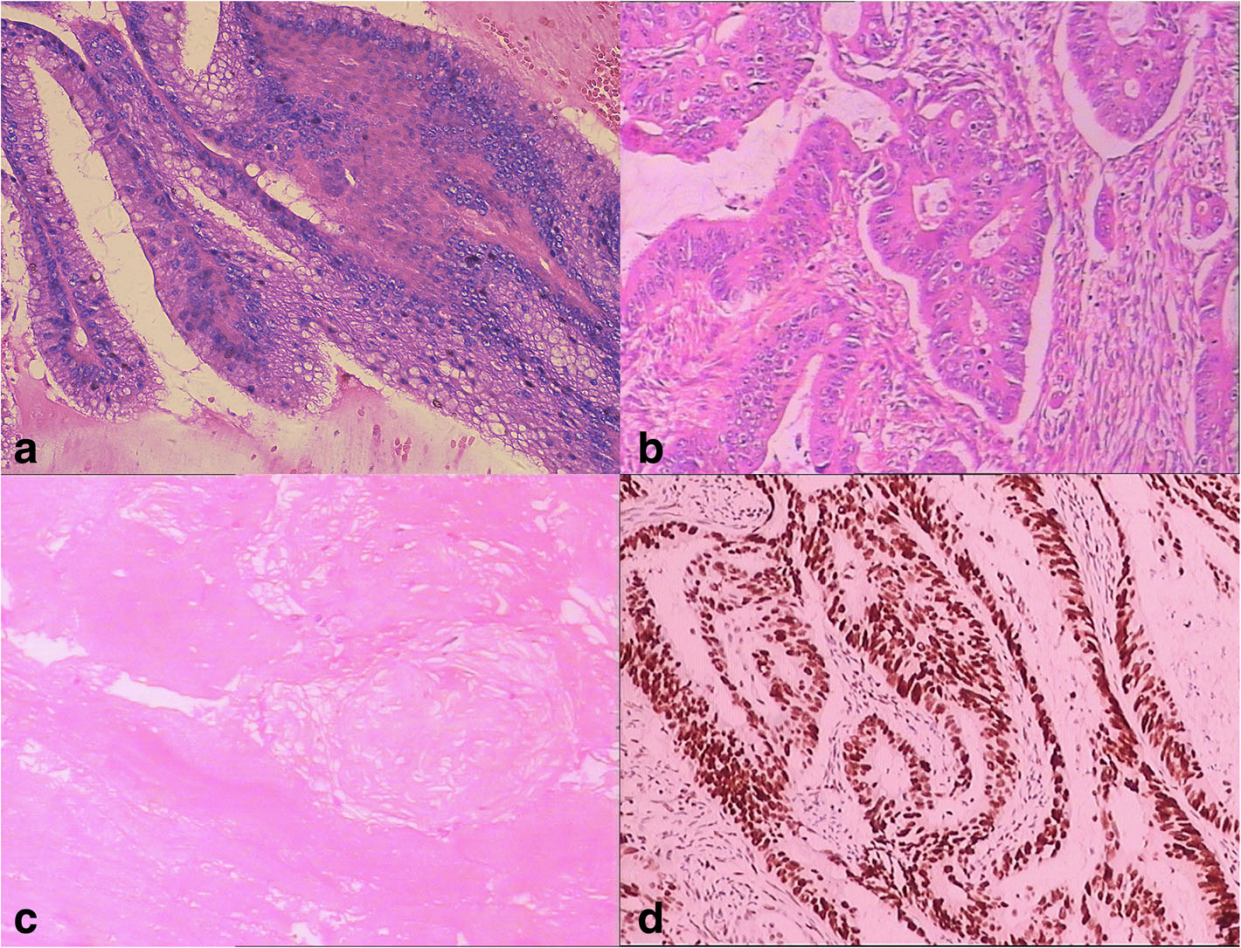
Postoperative histological pathologic diagnoses were appendiceal mucinous neoplasm, rectal cancer and PMP. The rectal cancer was medium differentiated adenocarcinoma (**a**). The appendiceal mass was low-grade appendiceal mucinous neoplasm (**b**). Large quantities of cavities containing mucus were observed in the fibrous tissue (**c**). Immunohistochemical staining of the rectal tumour revealed the following staining results: PTEN (++), ERRCC1 (++), VEGF (++), TS (−), EGFR (++), HER2 (0), PMS2 (+), MLH1 (++), MSH2 (+++), MSH6 (+++), and MGMT (+) (**d**)

The postoperative histological pathologic diagnoses were appendiceal mucinous neoplasm, rectal cancer and PMP. The rectal cancer was a medium differentiated adenocarcinoma, approximately 50% of which was a mucinous adenocarcinoma. Serosa invasion, intestinal ulcerations and perineural invasion were noted, but vascular invasion was not observed (Fig. 4a). In the appendiceal mass, a crowded glandular epithelium with mild nuclear abnormalities, including the pseudo-layer arrangement, was noted. The tumour was LAMN (Fig. 4b). Moreover, numerous cavities containing mucus were observed in the fibrous tissue (Fig. 4c). The immunohistochemical staining of the rectal tumour revealed the following: PTEN (++), ERRCC1 (++), VEGF (++), TS (−), EGFR (++), HER2 (0), PMS2 (+), MLH1 (++), MSH2 (+++), MSH6 (+++), and MGMT (+) (Fig. 4d).

Hyperthermic intraperitoneal chemotherapy (HIPEC) was not performed during the surgery because of disagreement among the patient’s family members. We strongly recommended that the patient receives chemotherapy or radiotherapy after surgery. However, to date, the patient did not receive these treatments due to economic difficulties. At the 1-year follow-up visit, no tumour recurrence was discovered by CT.

## Discussion and conclusion

### Incidence

PMP is a rare type of peritoneal secondary tumour. The incidence of PMP was initially proposed to be approximately 1 per million population per year [9]. The incidence of PMP has been estimated to be approximately 2 per million annually based on Smeenk’s research. According to experience at high volume centres, the actual incidence may be 3–4 operable cases per million per year [10]. Because limited data are available, the true incidence of PMP in the population is unknown [10–12]. PMP is difficult to diagnose preoperatively, and most diagnoses are confirmed by laparotomy or postoperative pathology [4].

### Pathophysiology

Generally, the primary lesion originates from an adenoma, mucinous appendiceal adenocarcinoma or an ovarian tumour. However, primary peritoneal PMP has also been described. Indeed, case reports describing PMP originating from nearly every abdominal organ, including the fallopian tube, pancreas, intestine, urachus, and stomach, have been published, although some origins are unclear [13–17]. With the recent development of molecular diagnostic techniques, the appendix has been confirmed as the main organ affected by PMP, whereas the ovary is generally a secondary site [18]. Mucinous appendicular adenocarcinoma was the origin of PMP in this case.

PMP metastasis mainly occurs via implanted metastasis rather than lymphatic metastasis or haematogenous metastasis. Dissemination occurs by the rupture of the primary lesion, which releases tumour cells into the abdominal cavity. Tumour cells produce mucin and are responsible for the development of the characteristic “jelly belly” [19, 20].

### Classification

The PMP histopathology and classification are confusing and challenging. PMP can be histologically divided into the following 3 types according to Ronnett’s classification: disseminated peritoneal adenomucinosis (DPAM), which tends to form benign lesions; peritoneal mucinous carcinomas (PMCA), which tends to form malignant tumours; and an intermediate type, which exhibits hybrid features of DPAM and PMCA [21]_._ DPAM is characterized by peritoneal lesions composed of abundant extracellular mucin containing scanty focally proliferative mucinous epithelium and small cytological atypia. PMCA is characterized by peritoneal lesions composed of more abundant mucinous epithelium with the architectural and cytologic features of carcinoma [22]. According to the WHO’s classification reported in 2010 evaluating the histogenesis, molecular genetic findings and clinical behaviour of PMP, PMP can be unanimously divided into low-grade and high-grade PMP. Low-grade PMP is characterized by mucin pools with low cellularity (< 10%), unremarkable cytology and a non-stratified cuboidal epithelium. High-grade PMP is characterized by mucin pools with high cellularity, moderate/severe cytologic atypia and a cribriform/signet ring morphology with desmoplastic stroma [23].

### Clinical features

PMP is often asymptomatic during the initial stages and classically presents with vague abdominal symptoms when marked disease burden is noted. Patients often do not recall any acute abdominal pain associated with the tumour rupture. With increasing mucous accumulation, PMP can manifest as nausea, vomiting, anaemia, fatigue, loss of appetite, weight loss, ascites and other nonspecific symptoms [24, 25]. Intestinal obstruction is occasionally noted [26]. Upon physical examination, PMP patients exhibit increased abdominal girth, negative shifting dullness, dough kneading sensation upon abdominal palpation, abdominal tenderness and abdominal mass [27–29].

### Image characteristics

PMP typically appears on ultrasound as a moderate amount of ascites containing septation and echoes, invasive parenchymal nodules and peritoneal masses. Serrated or scalloping changes around the liver, spleen, uterus and other abdominal organs are noted. One of the most specific signs is the presence of hypoechoic areas in the thickened peritoneum, which typically has a cake-like appearance on ultrasound [30–34].

High-frequency ultrasound can clearly show the pressure trace of the abdominal organs and occasionally reveal primary PMP lesions [35]. Colour Doppler ultrasonography can reveal branched or reticulate blood vessels passing through the mass [34, 36]. It is important for patients to undergo a cytological evaluation and ultrasound-guided biopsy, which is considered safe, simple and effective, to diagnose PMP preoperatively. The jelly-like mucinous material can be obtained with ultrasound-guided biopsy [37, 38]. In this case, the initial diagnosis of suspected PMP depended on the characteristic yellow, jelly-like mucus extracted though ultrasound-guided biopsy and exfoliative cytology of the mucus.

CT has become the first choice because it can show the distribution and infiltrated range of the primary PMP lesion. An abnormal density of ascites is noted in CT scans of PMP patients. Other features include a shell-like pressure trace on the surface of the liver and spleen, omentum thickening, peritoneal infiltration and mesentery and grid-like changes [39–41]. The calcification in the low-density area is also a specific finding in pseudomyxoma peritonei [39]. When severely invaded by the tumour, the omentum will become a large piece of soft tissue in front of the intestine and typically appears as a density shadow called “omental cake”. The small intestine can exhibit “cloak sign” in the CT reconstruction due to considerable mucus ascites extruding into one side or both sides of the spine [42–44]. The above signs represent characteristic CT features of PMP and are diagnostically helpful.

Magnetic resonance imaging (MRI) can show similar characteristics as CT, but MRI identifies the intestinal wall and the tumour’s boundary. Multi-directional MRI shows the relationship between the tumour and organ involvement, representing another advantage of MRI [45–47]. PET-CT is most useful for predicting peritoneal dissemination and evaluating the pathologic grade and potential for complete cytoreduction preoperatively [48]. The laparoscopic technology used for selective biopsy during the pathological examination can also collect ascites to identify the tumour cells, thereby improving the diagnostic accuracy. If the tumour widely transplants without surgical indications, HIPEC could be immediately performed through a peritoneal catheter under the guidance of a laparoscope as further treatment [49, 50].

### Tumour markers

Currently, PMP has no specific tumour markers. CEA, CA199 and CA125 are useful for the auxiliary diagnosis of PMP and reflect the severity and prognosis of the disease [51, 52]. The postoperative survival time of patients negative for CEA, CA199 and CA125 was 2.6–fold higher than that of patients positive for the three tumour markers. The CA199-negative group not only received sufficient cytoreductive surgery more easily but also exhibited a significantly longer median time to recurrence than the CA199-positive group [48, 49, 53, 54]. Pirjo Nummela et al. found that pseudomyxoma peritonei tumour cells invariably express CEA and EpCAM. These authors propose that CEA and EpCAM could be exploited to develop targeted therapies against this malignancy [55]. Immunohistochemistry can aid in the diagnosis of PMP based on the following features: CK7 (+), CK20 (+), CDX2 (+), MUC2 (+), MUC5AC (+), ER (−) and PR (−) [56–59]. Next generation sequencing (GNAS) plays a prognostic role, and patients with GNAS mutations exhibited significantly poorer outcomes in terms of progression-free survival [60].

### Treatment

Recently, CRS combined with HIPEC has been recommended as a standard treatment for PMP [61–63]. The completeness of cytoreduction (CC) is assessed at the end of surgery. By measuring the diameter of the largest remaining tumour lesion, the operation can be categorized as CC0 cytoreduction (complete removal of all visible lesion), CC1 cytoreduction (the largest residual lesion is < 0.25 cm), CC2 cytoreduction (0.25 cm ≤ largest residual lesion < 2.5 cm) and CC3 cytoreduction (the largest residual lesion is ≥2.5 cm). CC0 and CC1 are considered complete cytoreduction (CCRS), which is one of the most important prognostic factors for PMP [64–67]. The peritoneal carcinomatosis index (PCI) is widely used to assess the extent of disease. The abdomen could be divided into 9 anatomical areas with 4 further areas of the small bowel (upper and lower jejunum and upper and lower ileum) according to this scoring system. The tumour is accurately assessed in each area, and a score of 0–3 is given to each area (0 for no tumour, 1 for nodules < 0.5 cm, 2 for nodules between 0.5–5 cm and 3 for nodules > 5 cm) [68]. Although complete excision is not possible in some cases, maximal tumour debulking still offers significant survival advantages and significant improvements in the quality of life. Once cytoreduction is complete, HIPEC should be delivered. The cytotoxic drugs for HIPEC include 5FU, mitomycin C, doxorubicin, irinotecan, and cisplatin. An ex vivo assessment of drug sensitivity in PMP provides prognostic information. PMCA is slightly more resistant to platinum and 5FU than PMCA intermediate or disseminated peritoneal adenomucinosis. Tumour cells from patients previously treated with chemotherapy were generally less sensitive than those from untreated patients. Among patients with complete CRS, progression-free survival tends to be associated with the sensitivity to mitomycin C and cisplatin. Prior research further suggests that HIPEC could be used as a therapeutic adjunct to CRS, and a pretreatment assessment of drug sensitivity could benefit the individualization of HIPEC [69]. Unfortunately, HIPEC was not performed during the surgery due to disagreement in the patient’s family.

Compared with perioperative systemic chemotherapy (SC), SC after surgery exhibits remarkable effects. SC has minimal significance for LAMN, but a patient with high-grade appendiceal mucinous neoplasm can receive curative treatment [70]. Whole abdominopelvic radiotherapy using intensity-modulated arc therapy should be considered a palliative treatment option for the management of patients with recurrent PMP [71]. A study investigated the life quality of PMP patients who were treated by CRS combined with HIPEC and found that 79% of patients reported that they would accept this combined therapy again because it improved their lives. Moreover, their life quality was not reduced as a result of repeated treatments [72].

### Prognosis

Regarding the outcome of patients treated with CRS and HIPEC, Terence C et al. investigated 2298 cases of PMP patients who were treated with CRS + HIPEC. The results revealed that the median overall survival time was 196 months (16.3 years). The median progression-free survival time was 98 months (8.2 years). The 3-year survival rate was 80%. The 5-year survival rate was 74%. The 10-year survival rate was 63%. The 15-year survival rate was 59%. The authors also reported the outcome of 242 patients treated with CRS without HIPEC and showed that the 5-year survival rate was 40%, while the 10-year survival rate was 27%. This research confirms that patients with PMP treated with CRS combined with HIPEC exhibit good long-term therapeutic outcomes and guarded prognoses [73].

PMP accompanied by rectal cancer is highly rare, and only 4 cases have been reported worldwide by 2018 [5–8]. Khaldi F et al. first reported Pseudomyxoma peritonei complicating cancer of the rectum in 1993 [5]. Saad-Hossne R et al. presented “Peritoneal pseudomyxoma associated with synchronic malignant mucinous neoplasias of the caecum, appendix and rectum. Case report and review of the literature” in 2007 [6]. Newman CM and Moran BJ reported pseudomyxoma peritonei presenting as recurrent rectal cancer in 2010 [7]. Pseudomyxoma anorectum was described by Wang S et al. in 2017 [8]. To the best of our knowledge, PMP induced by low-grade appendiceal mucinous neoplasm (LAMN) accompanied by rectal cancer has never been reported. Rectal cancer was not identified until the signs of low intestinal obstruction and obvious expansion of the colon were noted. Identifying the low intestinal obstruction, which is easily covered by paralytic intestinal obstruction, was key. On the one hand, the analysis of the paralytic intestinal obstruction revealed the accumulation of considerable mucus in the abdominal cavity, which caused severe alterations in neurological, body fluid and metabolic function, potentially causing paralytic ileus. On the other hand, acute complete obstruction caused by rectal cancer could also cause paralytic ileus due to the overexpansion of the intestine for an excessively long time.

This study presents a rare case of PMP induced by LAMN accompanied by rectal cancer. There are some typical characteristics of PMP in imaging features, clinical manifestation and treatments. A CT scan of the abdomen and pelvis and ultrasound-guided biopsy of the abdomen should be performed in patients with suspected PMP as soon as possible. It should also be emphasized that PMP is accompanied by rectal cancer in rare cases. Therefore, it is important to not overlook the possibility of rectal cancer.

## Abbreviations

CC: Completeness of cytoreduction
CRS: Cytoreductive surgery
CT: Computed tomography
DPAM: Disseminated peritoneal adenomucinosis
HIPEC: Hyperthermic intraperitoneal chemotherapy
LAMN: Low-grade appendiceal mucinous neoplasm
MRI: Magnetic resonance imaging
PMCA: Peritoneal mucinous carcinomas
PMP: Pseudomyxoma peritonei
SC: Systemic chemotherapy
